# Suicide in cancer patients: incidence and risk factors (literature review)

**DOI:** 10.1192/j.eurpsy.2023.234

**Published:** 2023-07-19

**Authors:** H. Abrebak, F. Z. Chamsi, A. Essafi, A. Taqui, S. Radi, A. El Ammouri

**Affiliations:** Psychiatric department, faculty of medicine of tangier, Tangier, Morocco

## Abstract

**Introduction:**

Suicide is considered an important public health problem in contemporary society. Over 800,000 deaths by suicide are estimated each year and the mortality rate is 11.4 per 100,000 people.

In people with cancer, depression is a high-prevalence disorder that affects patients’ ability to cope with illness, decreases treatment acceptance, prolongs hospitalization, reduces quality of life, and increases the risk of suicide. In turn, the diagnosis of cancer is a serious stressor, with many physical and psychological consequences, and is thought to be a risk factor for suicide.

**Objectives:**

This study aimed to perform a literature review on the incidence and risk factors of suicide in cancer patients

**Methods:**

the search for articles was carried out in the electronic scientific databases PubMed, ScienceDirect and Scopus. Variables studied included suicide rate, type of cancer, demographic characteristics, and signs and symptoms associated with suicide using the descriptors “suicide” and “cancer”.

**Results:**

42 articles were selected. As in the general population, the risk of suicide was higher in men with cancer than in women with cancer. Cancer patients aged 65 or older have a higher suicide rate than those under 65. Prostate, lung, pancreatic, bladder and colorectal cancers are the types most at risk for suicide. The first year after diagnosis carries a higher risk of completed suicide. Multiple risk assessment tools have been developed and are effective in identifying patients with depression or hopelessness, factors associated with a higher risk of suicide. However, there are no tools that can sensitively and specifically predict suicide.

**Conclusions:**

The incidence of suicide in a person diagnosed with cancer is approximately double the incidence of suicide in the general population. Early detection of depression in particular cancer populations, such as older male patients, can help identify those most at risk for suicide.

**Disclosure of Interest:**

None Declared

